# Correlation analysis of ApoB and TyG index levels with residual cardiovascular risk in patients with acute myocardial infarction

**DOI:** 10.3389/fendo.2025.1542190

**Published:** 2025-09-02

**Authors:** Yuanyuan Wang, Dachuan Guo, Youzhi Wang, Jianmin Yang, Peng Li

**Affiliations:** ^1^ Department of Emergency, Renmin Hospital, Hubei University of Medicine, Shiyan, Hubei, China; ^2^ State Key Laboratory for Innovation and Transformation of Luobing Theory, Chinese Ministry of Education, Chinese National Health Commission and Chinese Academy of Medical Sciences, Jinan, Shandong, China; ^3^ Key Laboratory of Cardiovascular Remodeling and Function Research, Chinese Ministry of Education, Chinese National Health Commission and Chinese Academy of Medical Sciences, Jinan, Shandong, China; ^4^ Department of Cardiology, Qilu Hospital of Shandong University, Jinan, Shandong, China; ^5^ Department of Emergency Medicine, The Affiliated Hospital of Qingdao University, Qingdao, Shandong, China

**Keywords:** ApoB, LDL-C, TyG index, acute myocardial infarction, coronary atherosclerosis, residual risk, adverse cardiovascular events

## Abstract

**Background:**

Low-density lipoprotein cholesterol (LDL-C) has now been the primary target for lipid-lowering therapy in the European and US guidelines for the management of dyslipidemia, with increasing interest in apolipoprotein B (ApoB) as a secondary target. The relationship between ApoB and the severity of acute myocardial infarction as well as residual risk still needs to be further determined. Coronary atherosclerosis occurs as a result of a complex set of factors, and there is a strong relationship between insulin resistance and cardiovascular disease. In contrast, there are limited studies on the relationship between TyG index (triglyceride glucose index), an indicator of insulin resistance, and cardiovascular disease. The purpose of this study was to investigate the value of ApoB and TyG index in assessing the severity of myocardial infarction and predicting prognosis.

**Methods:**

This study included 712 participants with acute myocardial infarction for a 5-year follow-up. Spearman correlation analysis and generalized linear model analysis were used to assess the correlation between ApoB and the severity of coronary atherosclerosis. Risk regression analysis was used to assess the correlation between ApoB and residual risk in patients with acute myocardial infarction, and the C-statistic, net reclassification index (NRI), and integrated discriminant improvement index (IDI) were further calculated to assess the predictive value of ApoB for residual risk after myocardial infarction.

**Results:**

Categorizing apoB, LDL-C, and TyG indices according to tertiles, higher levels of ApoB were significantly associated with the severity of coronary artery stenosis in patients with acute myocardial infarction (*P*<0.001), whereas no such associations were found for elevated levels of LDL-C and TyG indices (*P >*0.05). Higher levels of apoB were significantly associated with residual risk of coronary atherosclerotic heart disease after full adjustment for confounders. Higher levels of ApoB were significantly associated with residual risk of coronary atherosclerotic heart disease by binary logistic regression analysis after complete adjustment for confounders. In multivariate variable-adjusted models, the OR and 95% CI for intermediate levels of ApoB (0.85-1.05 g/L) compared with low levels of ApoB (≤0.84 g/L) and residual risk after myocardial infarction was 2.06 (1.11, 3.81) (P<0.05), and for high levels of ApoB (≥1.06 g/L) the OR and 95% CI was 2.60 (1.29, 5.26) (P < 0.05); for each SD increase in ApoB level, the increase in residual risk after myocardial infarction would increase 4.75-fold (P = 0.001). Higher levels of TyG index were not found to be significantly associated with residual risk after myocardial infarction (P > 0.05). The inclusion of LDL-C, ApoB, and TyG indices in the constructed baseline risk model, and ApoB significantly improved the predictive ability of the traditional risk model for residual risk. The ROC curve of the baseline risk model showed an AUC of 0.649; the AUC after adding LDL-C to the model was 0.680 (*P*=0.05684); the AUC after adding TyG to the model was 0.663 (*P*=0.1635); and the addition of ApoB to the baseline model increased the AUC substantially to 0.702 (*P*= 0.00417). Inclusion of ApoB in the baseline risk model improved the prediction of MACE most significantly in the baseline model (net reclassification index [NRI]: 0.3324, *P* < 0.001; integrated discriminant improvement index [IDI]: 0.0414, *P* < 0.001); with the inclusion of LDL-C the NRI was 0.3218 (*P* < 0.001), and IDI was 0.0263 (*P* < 0.001); NRI after inclusion of TyG index was 0.2169 (*P* = 0.017); IDI was 0.0082 (*P* = 0.022).

**Conclusions:**

ApoB is an independent risk factor for major adverse cardiovascular events (MACE) following myocardial infarction. Elevated ApoB levels are more advantageous than elevated LDL-C levels in assessing the severity of coronary artery stenosis in myocardial infarction patients and predicting residual risk after myocardial infarction. Therefore, in patients with acute myocardial infarction, ApoB can be considered to guide further intensive treatment. However, the TyG index did not demonstrate a significant advantage in predicting cardiovascular residual risk in this study.

## Background

Abnormalities in lipid profiles are closely associated with cardiovascular disease (CVD), which remains the leading cause of death worldwide (1, 2). It is well known that low-density lipoprotein cholesterol (LDL-C) is an important risk factor for atherosclerosis as well as for the development and progression of coronary artery disease, and LDL-C is therefore used as a primary target for controlling cardiovascular risk by lowering lipid levels (3). However, a large number of clinical studies have shown that even when LDL-C is controlled below target levels, there is still a substantial unexplained residual cardiovascular risk (4, 5). This has led us to consider atherogenic lipoproteins other than LDL-C and to reconsider LDL-C levels as the only indicator of cardiovascular risk reduction.

2019 European Society of Cardiology/European Atherosclerosis Society Apolipoprotein B (ApoB) is a more accurate marker of cardiovascular risk than LDL-C or non-high-density lipoprotein cholesterol (non-HDL-C), according to prospective observational studies, Mendelian randomization analyses, and statin drug trials (6, 7). ApoB-containing lipoproteins include triglyceride-rich lipoproteins known as celiac microparticles (CM), very low-density lipoproteins (VLDL) and their individual remnants, cholesterol-rich low-density lipoproteins (LDL), and lipoprotein (a) [Lp(a)]. The ApoB family includes ApoB100 and ApoB48, with ApoB-100 being the VLDL, LDL, and Lp (a) major structural proteins, and ApoB-48 is the major and irreplaceable apolipoprotein of CM, so every atherogenic lipoprotein contains ApoB particles (8, 9). A prospective cohort study found that the risk of myocardial infarction was strongly associated with the amount of ApoB, independent of lipoprotein type and lipid content, in both primary and secondary prevention cohorts, and that ApoB may be a major driver of atherosclerosis (10). In addition, insulin resistance (IR) is an important factor in the pathogenesis of diabetes mellitus, and extensive studies have found that IR is also a potential risk factor for the development of CVD, as IR leads to atherosclerosis as well as small vessel disease (11, 12). The triglyceride-glucose index (TyG), a marker of insulin resistance, has been proposed as a reliable alternative to IR, so the relationship between TyG and CVD needs to be further investigated (13).

To investigate the correlation between the levels of ApoB and LDL-C and the severity of coronary atherosclerosis in patients with myocardial infarction, and to further investigate whether ApoB is superior to LDL-C in assessing the residual risk after myocardial infarction, as well as to understand the correlation of TyG with acute myocardial infarction and the prognosis, a retrospective cohort study was performed. In this study, we calculated the Gensini score based on invasive coronary angiography, which was utilized to assess the severity of coronary atherosclerosis; residual risk after myocardial infarction was assessed based on the presence or absence of Major adverse cardiovascular events (MACE).

## Methods

### Study design and population

**Figure 1:**
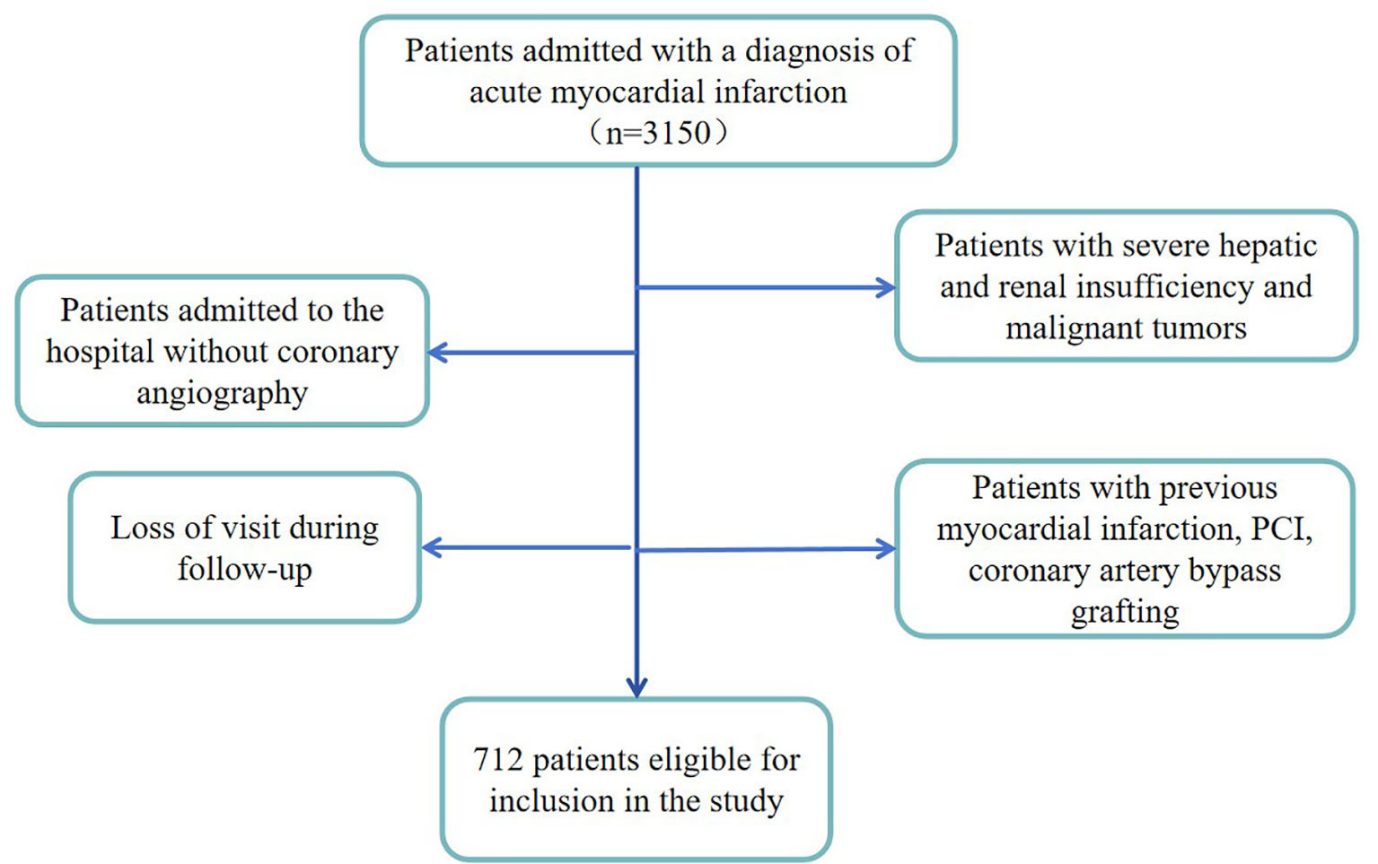
Flow diagram of participants enrolled.

We included 3150 patients with acute myocardial infarction in a retrospective cohort study in the hospital database between 2016.08-2019.2. We excluded patients who did not undergo coronary angiography after admission, patients with malignancy or severe hepatic or renal insufficiency, patients with a history of previous infarction, patients after previous coronary stent implantation or coronary artery bypass grafting, and patients who were lost to follow-up, resulting in a total of 712 patients enrolled in the study (**Figure 1**).

### Data acquisition and measurement

Relevant basic data including gender, age, height, weight, BMI, smoking history, history of hypertension, and history of diabetes mellitus were captured through e-case. Hypertension was defined as taking antihypertensive medication, having a systolic blood pressure ≥140 mm Hg or diastolic blood pressure ≥90 mm Hg (14). Diabetes mellitus was defined as taking antidiabetic medication, injecting insulin, having a fasting blood glucose ≥7.0 mmol/L, a 2-hour plasma glucose (PG) ≥200 mg/dl (11.1 mmol/L), or a glycosylated hemoglobin (HbA1c) ≥6.5% (15).Fasting venous blood samples were drawn and fasting blood glucose, apolipoprotein B (ApoB), cholesterol (TC), low-density lipoprotein cholesterol (LDL-C), high-density lipoprotein cholesterol (HDL-C), triglycerides (TG), and lipoprotein (a) [Lp(a)] were measured over a period of 2 hours and the triglyceride-glucose (TyG) index was derived from calculations using the following formula:TyG= Triglycerides (mg/dl)*plasma glucose (mg/dl)/2] (16). Each biochemical level is routinely measured by clinical laboratories in hospital. TyG is used to assess insulin resistance and to predict cardiovascular risk, and some studies have confirmed a positive correlation between TyG and the prevalence and prognosis of cardiovascular risk in the general population (17). Invasive coronary angiography was performed by experienced interventional cardiologists, who were unaware of the subject’s clinical information.

The Gensini score is calculated by assigning a score to the severity of stenosis in each coronary artery as follows: ≤25% stenosis is scored as 1, 26% to 50% stenosis is scored as 2, 51% to 75% stenosis is scored as 4, 76% to 90% stenosis is scored as 8, 91% to 99% stenosis is scored as 16, and total occlusion is scored as 32. Subsequently, each lesion score was multiplied by a factor that took into account the importance of the location of the lesion in the coronary circulation (5 for the left main coronary artery, 2.5 for the proximal left anterior descending coronary artery, 2.5 for the proximal left anterior descending coronary artery). 5 for the proximal segment of the rotary artery, 1.5 for the middle segment of the left anterior descending coronary artery, 1.0 for the right coronary artery, the distal segment of the left anterior descending coronary artery, the posterior collateral arteries, and the obtuse marginal artery, and 0.5 for the other segments). Finally, the Gensini score was calculated by summing the scores of the individual coronary segments (18).

### Follow-up and study endpoint

Patients were followed up as outpatients for 5 years after PCI. Residual risk after myocardial infarction was assessed primarily by the occurrence of MACE during the follow-up period, including all-cause death, recurrent myocardial infarction, ischemic stroke, readmission for heart failure, in-stent restenosis, or thrombosis. All-cause death was defined as death from cardiac or noncardiac causes and was only collected during the follow-up period. Myocardial infarction was diagnosed according to WHO criteria: typical symptoms plus ECG changes or elevated cardiac enzymes.

### Statistical analysis

Statistical analyses were performed using SPSS version 26.0 and R version 4.4.1. Differences were considered significant at *P* < 0.05.The grouping of ApoB and LDL-C levels was based on the tertile method. ApoB grouping: low level group ≤0.84 g/L, intermediate level group 0.85-1.05 g/L, and high level group ≥1.06 g/L; LDL-C grouping: low level group ≤2.38 mmol/L LDL-C group: low level group ≤2.38mmol/L, medium level group 2.39-3.09mmol/L, high level group ≥3.10mmol/L. Mean ± standard deviation was used to describe continuous variables with normal distribution and categorical variables were described by percentages. Comparisons between groups of continuous variables that conformed to normal distribution were performed using analysis of variance (ANOVA), and differences between groups were analyzed using the Kruskal-Wallis rank sum test for continuous variables that did not conform to normal distribution. Comparisons between groups were made using the chi-square test for categorical variables. After adjusting for covariates, i.e., age, sex, diabetes mellitus, hypertension, smoking status, cholesterol, and lipoprotein a, the associations of ApoB and LDL-C with the severity of coronary atherosclerosis (as assessed by the Gensini score) were assessed using Spearman correlation analysis and generalized linear modeling analyses. There was no multicollinearity between ApoB or LDL-C and the adjusted covariates. covariates (multicollinearity was defined as a correlation coefficient r ≥ 0.8 between variables).

Logistic regression analyses were used to assess the association of ApoB, LDL-C, and TyG indices with residual risk of atherosclerosis after PCI in patients with acute myocardial infarction. To further assess model performance, analysis was performed using subject work characteristics (ROC) curves and the area under the curve (AUC) measured by the C statistic was calculated to quantify the predictive ability of the model for residual risk. Tests of variability in AUC between models were assessed using the DeLong test. In addition, the net reclassification index (NRI) and the integrated discriminant improvement index (IDI) were further calculated to further assess the additional predictive value of ApoB, LDL-C, and TyG over and above residual risk factors after myocardial infarction.

## Results

### Baseline characteristics of the patients studied

A total of 712 AMI patients (mean age 59.6 years, of whom 63.06% were male) who underwent PCI were included in this study, and the mean concentrations of ApoB and LDL-C at baseline were categorized as 0.95 ± 0.27 g/L and 2.85 ± 0.93 mmol/L. The mean value of TyG at baseline was 1.49 ± 0.74. Study participants were categorized into low, intermediate, and high level groups based on ApoB levels, and the baseline characteristics of their subgroups are shown in **Table 1**. No statistical differences were found in baseline characteristics, that is, age, gender, hypertension, diabetes, and body mass index, according to ApoB levels; statistically significant differences were found between subgroups with different levels of ApoB in terms of smoking status, cholesterol, triglycerides, lipoprotein a, LDL cholesterol, HDL cholesterol, and metabolic index TyG. Moreover, the incidence of MACE and the mean Gensini score were higher in the high-level ApoB group than in the low-level group, and this difference was statistically significant.

**Table 1:** Baseline characteristics of participants with PCI according to ApoB concentrations.

Variables		ApoB concentration (g/L)			
	Total	≤0.84	0.85-1.05	≥1.06	*P*
Baseline characteristics					
Age (years)	59.59 ± 11.34	60.11 ± 11.11	60.03 ± 11.22	58.58 ± 11.68	0.386
Sex					0.531
Female	263 (36.9%)	95 (38.5%)	80 (34%)	88 (38.3%)	
Male	449 (63.1%)	152 (61.5%)	155 (66%)	142 (61.7%)	
Diabetes					0.408
Yes	166 (23.3%)	57 (23.1%)	49 (20.9%)	60 (26.1%)	
No	546 (76.7%)	190 (76.9%)	186 (79.1%)	170 (73.9%)	
Hypertension					0.718
Yes	403 (56.6%)	143 (57.9%)	128 (54.5%)	132 (57.4%)	
No	309 (43.4%)	104 (42.1%)	107 (45.5%)	98 (42.6%)	
Smoking Status					0.045
Yes	361 (50.7%)	141 (57.1%)	112 (47.7%)	108 (47.0%)	
No	351 (49.3%)	106 (42.9%)	123 (52.3%)	122 (53.0%)	
Overweight					0.591
Yes	475 (66.7%)	164 (66.4%)	152 (64.7%)	159 (69.1%)	
No	237 (33.3%)	83 (33.6%)	83 (35.3%)	71 (30.9%)	
Lipids					
TC (mmol/L)	4.72 ± 1.18	3.81 ± 0.69	4.63 ± 0.59	5.79 ± 1.19	<0.001
TG (mmol/L)	1.80 ± 1.57	1.61 ± 1.79	1.66 ± 1.15	2.16 ± 1.64	<0.001
HDL-C (mmol/L)	1.14 ± 0.26	1.11 ± 0.26	1.14 ± 0.26	1.18 ± 0.26	0.002
Lp (a) (mg/L)	241.09 ± 248.87	198.02 ± 191.46	249.61 ± 292.40	278.64 ± 249.14	<0.001
LDL-C (mmol/L)	2.85 ± 0.93	2.10 ± 0.47	2.82 ± 0.49	3.68 ± 0.96	<0.001
TyG	1.49 ± 0.74	1.31 ± 0.77	1.44 ± 0.66	1.74 ± 0.73	<0.001
Gensini Score	57.01 ± 37.06	44.09 ± 24.73	56.59 ± 30.65	71.31 ± 31.52	<0.001
MACE					0.001
Yes	153 (21.5%)	32 (13%)	52 (22.1%)	69 (30%)	
No	559 (78.5%)	215 (87%)	183 (77.9%)	161 (70%)	

Data are expressed as mean ± standard deviation, percentage.

ApoB levels were categorized into low (≤0.84), medium (0.85-1.05), and high (≥1.06) level groups.

BMI is body mass index (kg/m^2).

### Correlation between ApoB levels and severity of myocardial infarction

**Figure 2:**
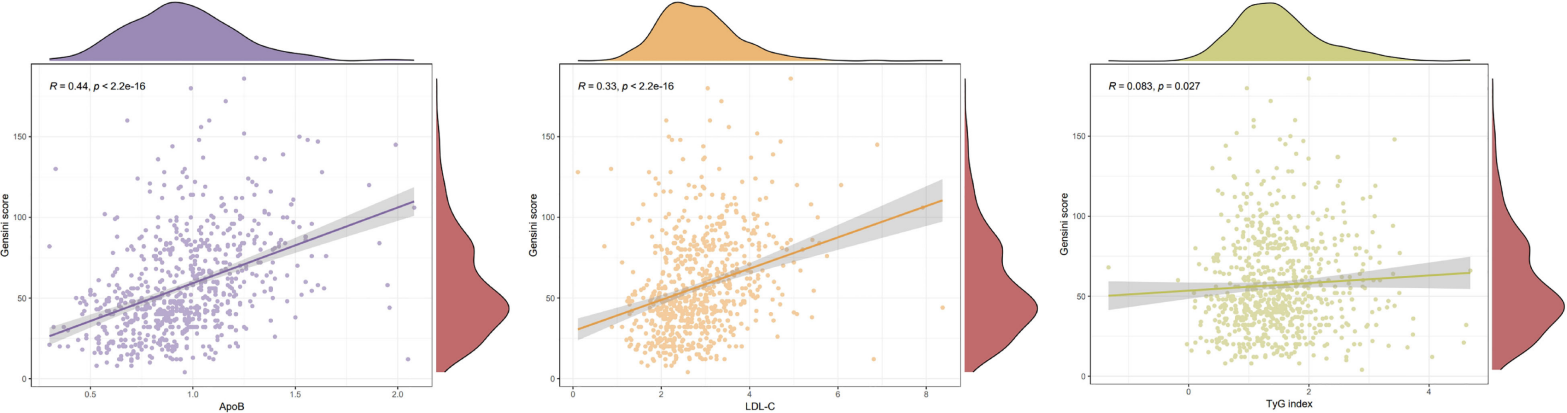
Scatter plot of spearman correlation analysis of ApoB, LDL-C, and TyG index with gensini score.

According to Spearman correlation analysis, the correlation coefficient between LDL-C level and Gensini Score was 0.33 (*p* < 0.001), the correlation coefficient between ApoB level and Gensini Score was 0.44(*p* < 0.001),and the correlation coefficient between TyG index and Gensini score was 0.083 (*p*=0.027) (**Table 2**). The correlation coefficients between the level of ApoB as well as the level of LDL-C and the degree of stenosis of coronary artery in myocardial infarction were positively correlated. positively correlated. The correlation between ApoB and LDL-C levels and the degree of stenosis was closer than that between ApoB and LDL-C levels. **Figure 2** is a scatter plot of ApoB, LDL-C, and TyG indices versus Gensini scores, which visualizes the positive correlation between ApoB and Gensini scores more clearly.

**Table 2:** Correlation analysis.

research indicators	r	*P*
ApoB (g/L)	0.44	<0.001
LDL-C (mmol/L)	0.33	<0.001
TyG	0.083	0.027

r is the Pearson correlation coefficient; Correlation analysis of ApoB level, LDL-C level, TyG and Gensini Score.

To further analyze the correlation between ApoB level, LDL-C level and the degree of coronary artery stenosis, we performed a generalized linear model analysis. The results showed that, after adjusting for sex, age, hypertension, diabetes mellitus, smoking history, BMI, cholesterol, HDL-C, triglycerides, and lipoprotein a, higher ApoB levels were significantly correlated with the severity of coronary artery stenosis (*P*<0.005), whereas higher levels of LDL-C as well as TyG did not have a significant correlation with coronary artery stenosis severity (*P*=0.160and *P*=0.196) (**Table 3**).

**Table 3:** B (95%CI)for the levels of ApoB, LDL-C and TyG.

Model 1				
ApoB (g/L)	≤0.84	0.85-1.05	≥1.06	*P*
	1	13.21 (6.78, 19.64)	25.67 (17.90,33.45)	<0.001
LDL-C (mmol/L)	≤2.38	2.39-3.09	≥3.10	*P*
	1	-2.71 (-8.96,3.54)	2.53 (-5.10,10.17)	0.234
TyG	≤1.11	1.12-1.68	≥1.69	*P*
	1	-1.20 (-6.48,4.09)	-1.69 (-7.09,3.71)	0.820
Model 2				
ApoB (g/L)	≤0.84	0.85-1.05	≥1.06	*P*
	1	13.27 (6.91,19.63)	25.98 (18.27,33.70)	<0.001
LDL-C (mmol/L)	≤2.38	2.39-3.09	≥3.10	*P*
	1	-2.00 (-8.25,4.25)	4.14 (-3.68,11.95)	0.160
TyG	≤1.11	1.12-1.68	≥1.69	*P*	
	1	-3.23 (-8.63,2.16)	-5.48 (-11.49,0.53)	0.196

B is the regression coefficient for the generalized linear model analysis; Model1: no adjustment for covariates; Model2: adjusted for age in years, sex, BMI, hypertension, diabetes mellitus, smoking history, HDL-C, lipoprotein a.

### Relationship between ApoB and residual risk after myocardial infarction

**Figure 3:**
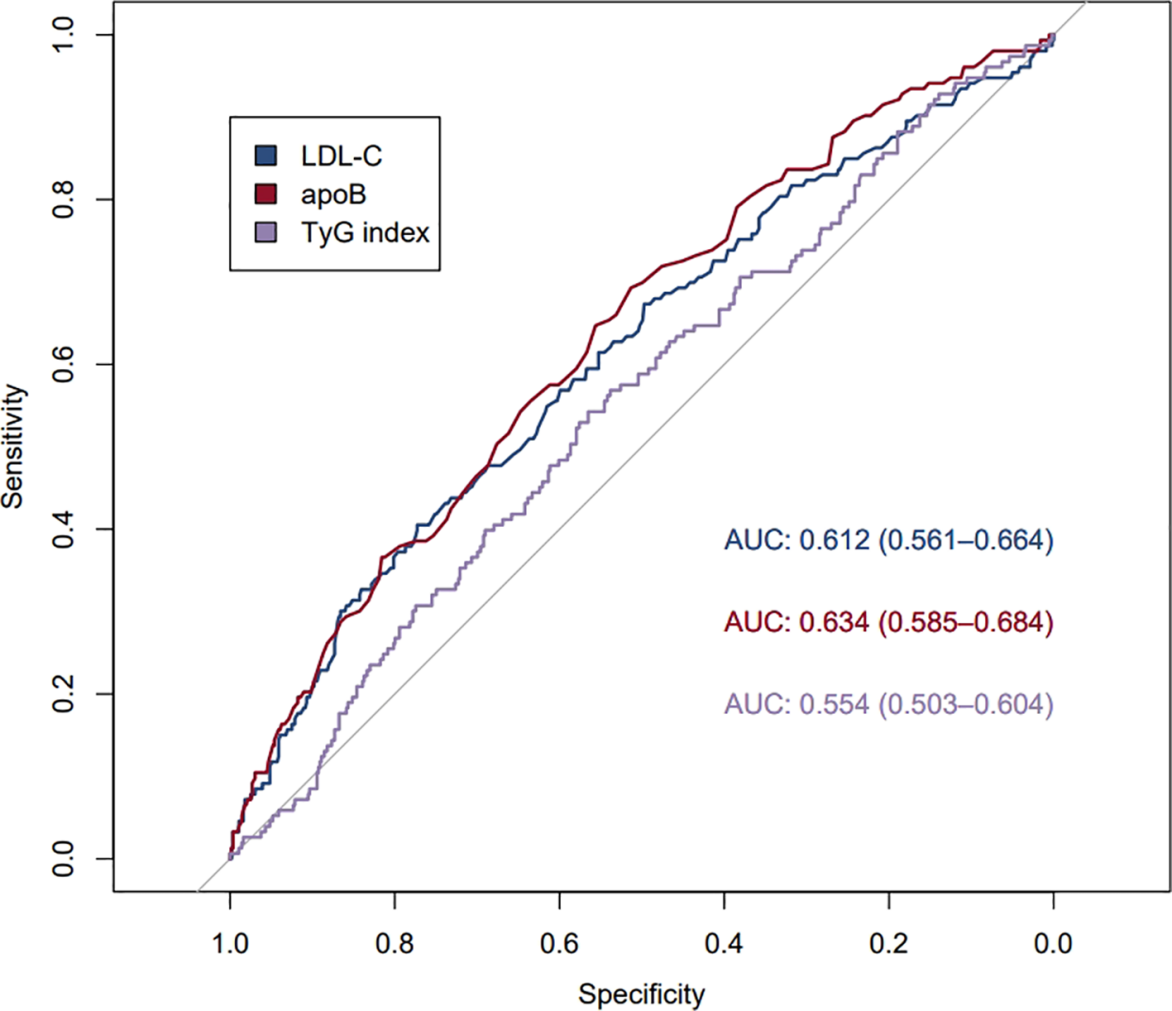
ROC curves for ApoB, LDL-C, and TyG.

A total of 153 patients experienced major adverse cardiovascular events during the 5-year follow-up period. By binary logistic regression analysis, higher levels of ApoB were significantly associated with the occurrence of adverse cardiovascular events after myocardial infarction after adjustment for multifactorial variables (**Table 4**). In the multivariate variable-adjusted model, the OR and 95% CI of intermediate levels of ApoB (0.85-1.05 g/L) compared with low levels of ApoB (≤0.84 g/L) and residual risk after myocardial infarction was 2.06 (1.11,3.81) (P<0.05), and the OR and 95% CI of high-levels of ApoB (≥1.06 g/L) associated with the occurrence of adverse cardiovascular events after myocardial infarction with an OR and 95% CI of 2.60 (1.29,5.26) (P<0.05) (**Table 4**); the risk of adverse cardiovascular events after myocardial infarction would increase 4.75-fold for each SD increase in ApoB levels (P=0.003) (**Table 4**). However, higher levels of TyG index were not found to be significantly associated with the occurrence of adverse cardiovascular events after myocardial infarction (P>0.05) (**Table 4**). Plotting the ROC curves showed an AUC of 0.634 for ApoB, 0.554 for TyG, and 0.612 for LDL-C, (**Figure 3**).The level of ApoB was a better predictor of the occurrence of major adverse cardiovascular events after myocardial infarction compared with the LDL-C and TyG indices (**Figure 3**).

**Table 4:** OR (95% CI) of different levels of ApoB, LDL-C, and TyG and adverse cardiovascular events after myocardial infarction.

ApoB (g/L)	≤0.84	0.85-1.05	≥1.06	*P*	Per one-unit increase
Model 1	1	1.88 (1.04,3.42)	2.36 (1.20,4.67)	0.002	5.58 (1.84,16.95)
Model 2	1	2.00 (1.08,3.68)	2.60 (1.29,5.25)	0.002	5.83 (1.88,18.10)
Model 3	1	2.06 (1.11,3.81)	2.60 (1.29,5.26)	0.003	5.75 (1.84, 18.00)
LDL-C (mmol/L)	≤2.38	2.39-3.09	≥3.10	*P*	Per one-unit increase
Model 1	1	0.89 (0.50,1.58)	1.22 (0.64,2.33)	0.941	1.01 (0.74,1.38)
Model 2	1	0.88 (0.49,1.58)	1.14 (0.58,2.21)	0.835	1.03 (0.75,1.42)
Model 3	1	0.84 (0.47,1.53)	1.12 (0.56,2.22)	0.986	1.00 (0.73,1.39)
TyG	≤1.11	1.12-1.68	≥1.69	*P*	Per one-unit increase
Model 1	1	0.94 (0.59,1.50)	1.20 (0.76,1.89)	0.603	1.07 (0.83,1.38)
Model 2	1	0.89 (0.54,1.45)	1.22 (0.73,2.05)	0.479	1.11 (0.83,1.50)
Model 3	1	0.88 (0.54,1.45)	1.25 (0.74,1.86)	0.336	1.16 (0.86,1.58)

Model 1: unadjusted for variables.

Model 2: adjusted for age, sex, BMI, hypertension, diabetes, smoking history.

Model 3: further adjusted for lipid variables (cholesterol, HDL-C, lipoprotein a), because cholesterol was severely multicollinear with ApoB and LDL-C, and therefore Model 3 did not include cholesterol.

### Incremental effect of ApoB, LDL-C, and TyG indices in predicting residual risk after myocardial infarction

**Figure 4:**
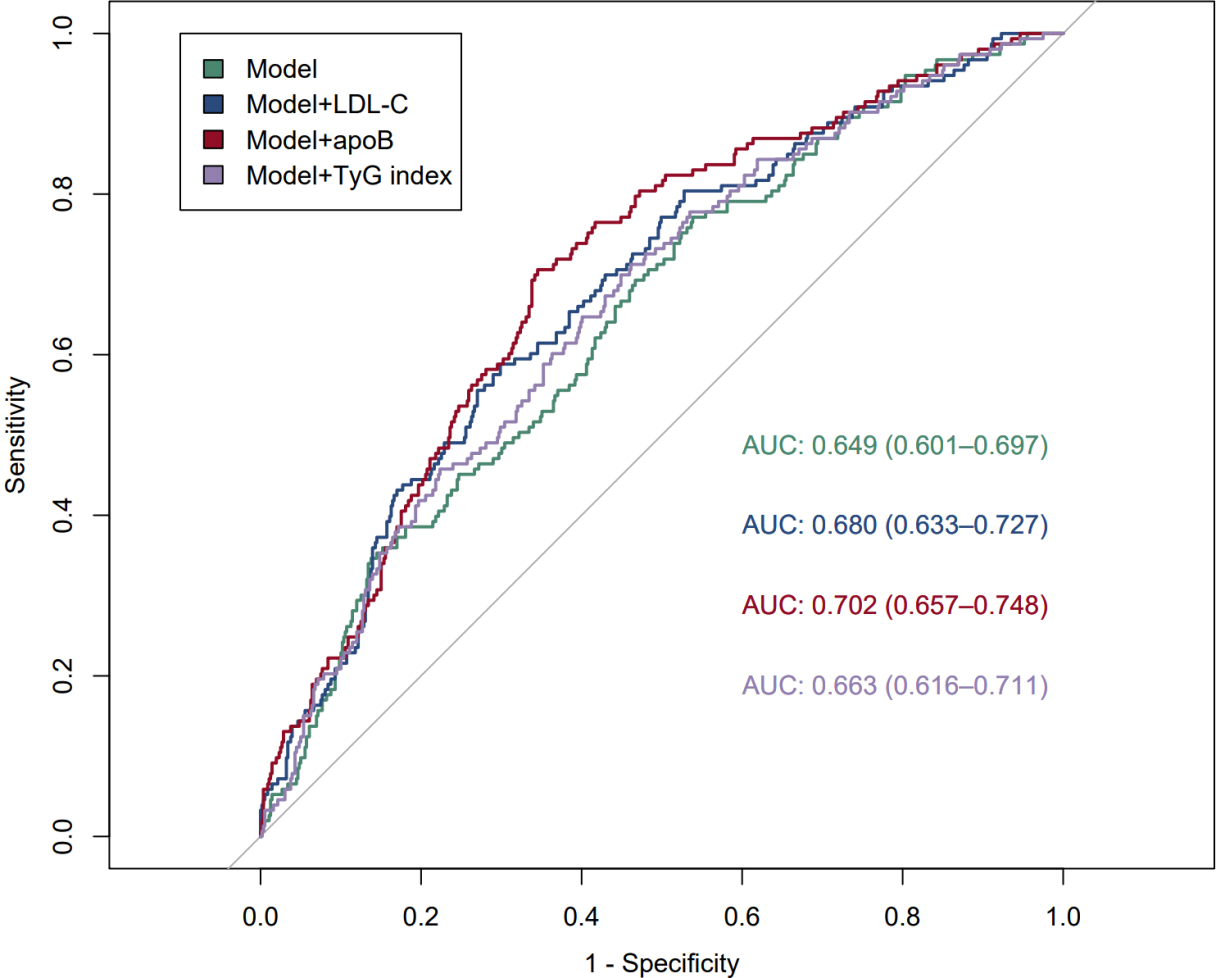
Working curve for subjects assessing ApoB, LDL-C, and TyG for prediction of adverse cardiovascular events after myocardial infarction model: refers to baseline risk modelling.

In the risk analysis of adverse cardiovascular events in patients with acute myocardial infarction, ROC curves were constructed to assess the predictive ability of the baseline risk model and the baseline risk model plus the LDL-C, ApoB, and TyG indices to predict the occurrence of adverse cardiovascular events after myocardial infarction (**Figure 4**). The baseline risk model included sex age, gender, BMI, hypertension, diabetes mellitus, smoking history, HDL-C, and lipoprotein a. The ROC curves for the baseline risk model showed a baseline AUC of 0.649 (0.601-0.697); the AUC after the addition of LDL-C to the model was 0.680 (P=0.057); and the AUC after the addition of TyG index to the model was 0.663 (P=0.164); the addition of ApoB to the baseline model AUC increased substantially to 0.702 (P=0.004) (**Figure 4**). In patients included in the study, improvements in the predictive ability of the baseline risk model were observed by including ApoB, LDL-C, and the TyG index in the baseline risk model (**Table 5**). In contrast to the addition of LDL-C or TyG indices (net reclassification index [NRI] for the inclusion of LDL-C: 0.3218, P < 0.001; integrated discriminant improvement index [IDI] for the inclusion of LDL-C: 0.0263, P < 0.001; net reclassification index [NRI] for the inclusion of TyG indices: 0.2169, P = 0.017; and net reclassification index [NRI] for the inclusion of the TyG index with Integrated Discriminant Improvement Index [IDI]: 0.0082, P=0.022), and the inclusion of ApoB in the baseline risk model improved the prediction of MACE in the baseline model most significantly (net reclassification index [NRI]:0.3324, P<0.001; Integrated Discriminant Improvement Index [IDI]: 0.0414, P<0.001) (**Table 5**). By comparing the INR as well as the IDI, the ApoB, LDL-C, and TyG indices all had an incremental effect on the predictive ability of the baseline model, but the incremental effect of ApoB was the most significant.

**Table 5:** Incremental effect of adding ApoB, LDL-C, and TyG indexes to baseline risk models for predicting adverse cardiovascular events after myocardial infarction.

Model	C-statistic	P-value	NRI (95%CI)	P-value	IDI (95%CI)	P-value
Baseline Model	0.649 (0.601-0.697)		Ref		Ref	
+LDL-C	0.680 (0.633-0.727)	0.05684	0.3218 (0.1445- 0.499)	<0.001	0.0263 (0.0119- 0.0406)	<0.001
+ApoB	0.702 (0.657-0.748)	0.00417	0.3324 (0.1553- 0.5095)	<0.001	0.0414 (0.0236-0.0592	<0.001
+TyG index	0.663 (0.616-0.711)	0.1635	0.0.2169 (0.0388 - 0.395)	0.017	0.0082 (0.0012 - 0.0152)	0.022

## Discussion

Our study provides insight into the value of ApoB in assessing the degree of coronary artery stenosis in patients with myocardial infarction and in predicting residual risk after myocardial infarction. 1) In the participating patients with acute myocardial infarction, higher levels of ApoB were significantly associated with the degree of coronary artery stenosis and residual risk after myocardial infarction in the participants, whereas no correlation was found for LDL-C and TyG. 2) After adjusting for confounders, ApoB was an independent risk factor for residual risk after myocardial infarction, with higher levels of ApoB associated with a higher risk of adverse cardiovascular events. 3) Adding ApoB to established baseline risk models showed the most significant incremental impact on predicting residual cardiovascular risk compared with other metrics. Early identification of residual risk after myocardial infarction is essential for the prevention of adverse cardiovascular events. Our results suggest that ApoB appears to be superior to LDL-C in assessing the degree of coronary artery stenosis as well as residual risk in patients after myocardial infarction, and thus lowering ApoB levels could be valuable in further reducing residual cardiovascular risk.

The most probable explanation for the findings is that ApoB causes atherosclerosis by triglyceride-rich lipoproteins, namely, celiac microparticles (CM), very low-density lipoproteins (VLDL) and their respective residues, cholesterol-rich low-density lipoproteins (LDL), and lipoprotein (a) (19). The atherosclerosis of the atherosclerosis of the arteries is caused by ApoB-48, a triglyceride-rich lipoprotein, and ApoB-100, a triglyceride-rich lipoprotein. Fatty acids ingested by the body combine with ApoB-48 in the intestinal epithelial cells to form celiac particles, which are secreted into the circulation to be broken down by enzymes, and are metabolized in the liver to produce triglycerides, which combine with ApoB-100 to form VLDL, which are further broken down and metabolized, and progressively converted to IDL and LDL. As for triglycerides, the EAS Consensus of 2021 states that, when the TG> 1.2 mmol/L, TRL residues begin to accumulate in the blood, and TRL residues with diameters smaller than about 70 nm can enter the vascular subendothelium through trans-endothelial cytosis, and lipoproteins are retained and accumulated in the arterial wall, forming smaller and denser LDL with higher densities and smaller sizes, which has a stronger ASVD-causing effect. Thus, the use of LDL-C as an indicator of cardiovascular risk ignores the atherogenic potential of triglyceride-rich lipoproteins as well as lipoprotein (a). Concentrations of ApoB are directly proportional to the total number of atherogenic lipoprotein particles, so ApoB may be a better predictor than LDL-C.

Comparable results have been reported in previous studies of participants treated with statins. In 13,015 patients treated with statins in the Copenhagen Total Population Study, elevated concentrations of apolipoprotein B and non-high-density lipoprotein cholesterol were associated with an increased risk of all-cause mortality and myocardial infarction, whereas elevated concentrations of low-density lipoprotein cholesterol were not associated with an increased risk of all-cause mortality and myocardial infarction (3). Likewise, Marston et al. found in a cohort study that the strongest association with MI was captured by the amount of ApoB-containing lipoproteins, independent of lipid content (cholesterol or TG) or type of lipoprotein (LDL or TG-rich), in a primary prevention cohort that did not receive statin therapy and in a secondary prevention cohort that did, and that ApoB-containing lipoprotein particles that carried the amount of lipid carried on ApoB-containing lipoprotein particles does not pose an additional risk of exceeding the ApoB concentration (10). In multivariate Mendelian randomization analyses, the associations of triglyceride and LDL-C levels with risk of coronary heart disease became null after adjustment for ApoB (20). In previous studies, which were not conducted in Chinese, our study found that ApoB was superior to LDL-C in assessing the severity of myocardial infarction and residual risk.

In previous studies, few studies have been conducted in Chinese populations, and of those that have been conducted, the study population was untreated patients with coronary atherosclerotic heart disease, and only the correlation between lipid biomarkers and the degree of coronary artery stenosis was examined (21, 22).In a study of Chinese people, only 131 patients treated with statins were included in the study (23). The population of acute myocardial infarction was targeted and 712 participants were included in our study to determine the correlation between ApoB and LDL-C levels and coronary severity as well as prognosis, and the metabolic index TyG was also added to our study. We also developed risk models to further evaluate the predictive performance of lipid and metabolic indices on residual risk after acute myocardial infarction by using C-statistics, IDI, and NRI. In the Chinese population, many patients with coronary atherosclerotic heart disease have myocardial infarction as their first symptom, and they do not undergo health checkups or treatments before myocardial infarction occurs, so the study of acute myocardial infarction is more relevant.

The 2019 European Society of Cardiology/European Atherosclerosis Society guidelines emphasize the status of ApoB by suggesting that it should be routinely measured (24), whereas the guidelines in China, as well as in countries such as the United States, do not point this out. LDL-C remains the primary target for lipid-lowering therapy in the management of dyslipidemia in Europe and the United States (6, 25).Analyzing the reasons for this, there is ample evidence that lowering ApoB levels significantly reduces the risk of developing coronary heart disease as well as improves prognosis. However, the current threshold for ApoB as a risk modifier in patients with myocardial infarction is relatively underdeveloped and requires further study (3).The understanding of ApoB and atherosclerosis formation needs to be further developed, although there is evidence that trapping of particles of ApoB in the arterial wall is the underlying cause of atherosclerosis (8, 10).In our study, ApoB was an independent risk factor for MACE after acute myocardial infarction and was a better predictor of residual risk. Therefore, in patients receiving lipid-lowering therapy after myocardial infarction, the use of apoB may be considered to guide further intensive therapy and routine measurement of apoB may be recommended, even if LDL-C levels have been reduced to below standard.

Previous epidemiologic studies have confirmed that cholesterol, especially LDL cholesterol, and triglycerides are risk factors for cardiovascular disease and that elevated levels of both increase the risk of cardiovascular disease (26).However, despite lowering LDL-C as well as triglyceride levels to below standard or even lower, there is still a residual risk of cardiovascular disease, and thus cardiovascular disease may be caused by a complex set of factors (5, 27).A prospective cohort study found an increased risk of myocardial infarction with elevated baseline or long-term TyG levels (12).Therefore, our study incorporated the TyG index to further investigate its relationship with residual risk after myocardial infarction, and the results showed that the inclusion of the TyG index among the traditional risk factors had an incremental effect in predicting the occurrence of adverse cardiovascular events after myocardial infarction. However, the small effect may limit its value in clinical practice, and its clinical significance needs to be further investigated.

## Limitations

Regarding the limitations of this study, firstly, our study was a single-center retrospective cohort study; therefore, it is difficult to fully elaborate the causal relationship between ApoB and severity of myocardial infarction as well as the residual risk after myocardial infarction, and further prospective studies are needed in the future to validate our findings. Second, ApoB, LDL-C, and TyG levels were determined only at the time of admission, and the levels during follow-up were not further analyzed.

## Conclusions

ApoB is an independent risk factor for residual risk after myocardial infarction and, when combined with traditional risk indicators, can better predict cardiovascular residual risk. Therefore, ApoB has advantages in assessing the severity of coronary artery stenosis and residual risk in patients with acute myocardial infarction. In patients with acute myocardial infarction, ApoB can be considered to guide further intensive treatment. This has important implications for the management and treatment goals of lipid-lowering therapy in future clinical practice. While managing lipid levels, the TyG index, an indicator of insulin resistance, should also be actively controlled. Further reducing TyG index levels can lower the incidence of adverse cardiovascular events after myocardial infarction.

## Data Availability

The datasets used and/or analyzed during the current study are available from the corresponding author on reasonable request.
