# Correction: Correlation analysis of ApoB and TyG index levels with residual cardiovascular risk in patients with acute myocardial infarction

**DOI:** 10.3389/fendo.2025.1699149

**Published:** 2025-09-15

**Authors:** Yuanyuan Wang, Dachuan Guo, Youzhi Wang, Jianmin Yang, Peng Li

**Affiliations:** ^1^ Department of Emergency, Renmin Hospital, Hubei University of Medicine, Shiyan, Hubei, China; ^2^ State Key Laboratory for Innovation and Transformation of Luobing Theory, Chinese Ministry of Education, Chinese National Health Commission and Chinese Academy of Medical Sciences, Jinan, Shandong, China; ^3^ Key Laboratory of Cardiovascular Remodeling and Function Research, Chinese Ministry of Education, Chinese National Health Commission and Chinese Academy of Medical Sciences, Jinan, Shandong, China; ^4^ Department of Cardiology, Qilu Hospital of Shandong University, Jinan, Shandong, China; ^5^ Department of Emergency Medicine, The Affiliated Hospital of Qingdao University, Qingdao, Shandong, China

**Keywords:** ApoB, LDL-C, TyG index, acute myocardial infarction, coronary atherosclerosis, residual risk, adverse cardiovascular events

Affiliation “Department of Emergency Medicine, The Affiliated Hospital of Qingdao University, Qingdao, Shandong, China” was omitted for authors “Youzhi Wang and Peng Li”. This affiliation has now been added for author “Youzhi Wang and Peng Li”.

Author “Youzhi Wang and Peng Li” were erroneously assigned to the affiliation “Department of Emergency, Renmin Hospital, Hubei University of Medicine, Shiyan, Hubei, P. R. China”. This affiliation has now been removed for authors “Youzhi Wang and Peng Li”.

The original version of this article has been updated.

